# Baculovirus 25K hijacks host UAP56 to facilitate nuclear export of viral mRNA in insect cells

**DOI:** 10.1128/jvi.01248-25

**Published:** 2025-09-23

**Authors:** Sixuan Xiao, Huizhen Guo, Jiayi Liu, Lihua Wei, Qingqing Yang, Enyu Xie, Bingbing Wang, Qingyou Xia, Liang Jiang

**Affiliations:** 1 Integrative Science Center of Germplasm Creation in Western China (CHONGQING) Science City, Biological Science Research Center, Southwest University 26463, Chongqing, China; 2 Key Laboratory for Germplasm Creation in Upper Reaches of the Yangtze River, Ministry of Agriculture and Rural Affairs, Chongqing, China; Wageningen University & Research, Wageningen, Netherlands

**Keywords:** mRNA export, silkworm, UAP56, 25K, baculovirus

## Abstract

*Bombyx mori* nucleopolyhedrovirus (BmNPV) is a baculovirus that hijacks host genes to cause viral infections. UAP56 is highly conserved in different species. Previous studies have shown that UAP56 is involved in several viral infections. However, its role in insect-baculovirus interactions remains unknown. In this study, we aimed to identify which BmNPV proteins interact with host UAP56 and to characterize the associated mechanism underlying viral infection using a BmNPV-silkworm model. Our data indicated that CCT018159, an inhibitor of UAP56, could suppress the proliferation of BmNPV and that the addition of CCT018159 within 12 h post-infection had a significant protective effect on BmE cells. To identify the interacting viral proteins, recombinant UAP56-GST was constructed through prokaryotic expression for pull-down screening. Further, immunofluorescence, co-immunoprecipitation, and pull-down analyses demonstrated that the late viral protein, 25K, directly binds to UAP56. CCT018159 did not affect the co-localization of 25K and UAP56 but disrupted the interaction between them, resulting in significant upregulation of viral mRNA content in the nucleus and opposite trend in the cytoplasm. Overexpression of 25K and UAP56 caused a significant reduction in viral mRNA content in the nucleus and a significant increase in the cytoplasm. The addition of CCT018159 counteracted this effect. Overall, our data show that the baculovirus 25K protein hijacks host UAP56 to facilitate the nuclear export of viral mRNA to cause infections.

**IMPORTANCE:**

Nuclear export of viral mRNA is essential for viral proliferation. UAP56 is highly conserved among species and is involved in multiple viral infections. In this study, we found that the *Bombyx mori* nucleopolyhedrovirus 25K protein hijacks host UAP56 to facilitate viral mRNA nuclear export, and disruption of their interactions can inhibit viral proliferation. Our results provide novel insights into the mechanism of insect-baculovirus interaction and emphasize the important role that 25K plays in baculovirus infection. This research not only deepens our understanding of the transcription and translation mechanisms of baculoviruses but also provides potential targets for antiviral research.

## INTRODUCTION

Baculoviruses are enveloped, rod-shaped, double-stranded DNA viruses with a genome size of 80–180 kbp (1). They mainly infect insects and replicate in the host cell nucleus (2). Baculoviruses achieve efficient replication by regulating host cell mechanisms (3), and their genetic engineering vectors are widely used in recombinant protein production and the development of biocontrol technology (4). *Bombyx mori* nucleopolyhedrovirus (BmNPV) is a typical baculovirus with a genome size of 128 kb that encodes 143 genes. BmNPV is a major pathogen of silkworms and causes serious economic losses in sericulture (5).

Baculoviruses mainly infect insects through oral ingestion (6), and their life cycle involves two morphologically distinct virions: the budded virion (BV) and occlusion-derived virion (ODV) (7). After the virus invades the host cell, it hijacks host genes for transcription and translation. Baculovirus gene expression is strictly time-regulated and can be divided into four categories according to the transcription initiation time: immediate-early (<4 h post-infection [hpi]), delayed-early (5–7 hpi), late (8–18 hpi), and very late genes (>18 hpi) (8). This tightly regulated temporal program ensures the ordered progression of viral replication from DNA synthesis to progeny virion assembly and egress. Early gene expression activates viral DNA replication and late gene expression (9), followed by assembly to produce new virions. A total of 28 early and 78 late BmNPV genes have been identified (10–12). When a large number of late genes are transcribed, the nucleation and transport efficiency of viral mRNA affect the number of progeny viruses and the entire infection process.

Baculoviruses regulate the host in two ways. On the one hand, the virus inhibits the host antiviral signaling pathway to achieve immune escape. For example, baculoviruses induce Bmserpin2 to inhibit the polyphenol oxidase pathway-mediated melaninization response (13); BmNPV induces BmPGRP2-2 to inhibit PTEN expression, resulting in an increase in p-Akt levels and the inhibition of apoptosis and autophagy (14); BmNPV downregulates BmSpry, activates the ERK pathway, and promotes viral proliferation (15). On the other hand, viruses hijack host genes to participate in multiple processes, such as viral transcription, translation, and replication, and promote virus proliferation. For example, the capsid protein, VP39, promotes viral replication by binding to host phosphatase 38K and DNA-binding protein P6.9 (16, 17); E25, a key component of the ODV envelope, regulates viral DNA release and intranuclear replication by interacting with host membrane proteins and nucleoporins (18); the virus-encoded BmNPV-mir-1 and BmNPV-mir-3 enhance BmNPV infection by regulating expression of the output protein 5 cofactor, Ran, and viral P6.9, respectively (19, 20). A large number of studies on baculovirus-insect interactions have focused on the function of baculovirus genes through sequence mutations; however, few have investigated the host proteins involved in baculovirus interactions.

UAP56 (NCBI reference sequence NP_004631.1) is known as an ATP-dependent RNA helicase. As a multifunctional RNA metabolism regulator, UAP56 participates in various RNA metabolic processes in eukaryotic cells. Its functions span through transcription, splicing, and quality control. First, in terms of regulating the assembly and fidelity of pre-mRNA spliceosomes, UAP56 assists in the binding of U2 small nuclear ribonucleoproteins to SF3b complexes by binding to the branch site region of pre-mRNA to ensure the precise assembly of spliceosomes (21). Second, in terms of RNA quality control for transcriptional coupling, UAP56 dynamically interacts with RNA polymerase II to monitor the integrity of nascent RNA during transcriptional elongation (22). In addition, UAP56 is a core component of transcription/export complex (TREX). TREX is a large multiprotein complex involved in cellular bulk mRNA nuclear export (23). UPA56 is hijacked by multiple viruses during viral infection to achieve efficient replication (24). One of its core functions is to promote viral RNA splicing and transcription complex assembly; for example, influenza A virus directly recruits UAP56 through nuclear proteins (NPs) to form TREX-NP complexes, enhancing viral RNA polymerase activity and regulating mRNA nuclear export (25). In addition, UAP56 participates in the infection process by mediating the nucleocytoplasmic transport of viral mRNA. For example, human cytomegalovirus binds to UAP56 using the UL69 protein, bypassing the host splicing mechanism to drive the nuclear export of intron-free virus mRNA (26). Hepatitis B virus regulates viral RNA splicing and transport through HBx protein interactions and inhibits the interferon signaling pathway to maintain persistent infection (27). Evolutionary conservation studies have shown that the RNA transport function of UAP56 is highly conserved in eukaryotes, such as *Drosophila* and yeast, and the deletion of its homologous genes (Hel25E and yeast Sub2) leads to RNA retention in the nucleus, providing a molecular basis for the mechanism of cross-species host hijacking by viruses (28, 29).

We speculated that UAP56 may be involved in baculovirus-insect interactions, and its function during these interactions was investigated using the BmNPV-silkworm model. We examined the effect that UAP56 has on the proliferation of BmNPV, identified the viral proteins that interact with UAP56, and analyzed the molecular mechanism of action of UAP56 in viral infection.

## RESULTS

### CCT018159 targets UAP56 to inhibit BmNPV proliferation

**Fig 1:**
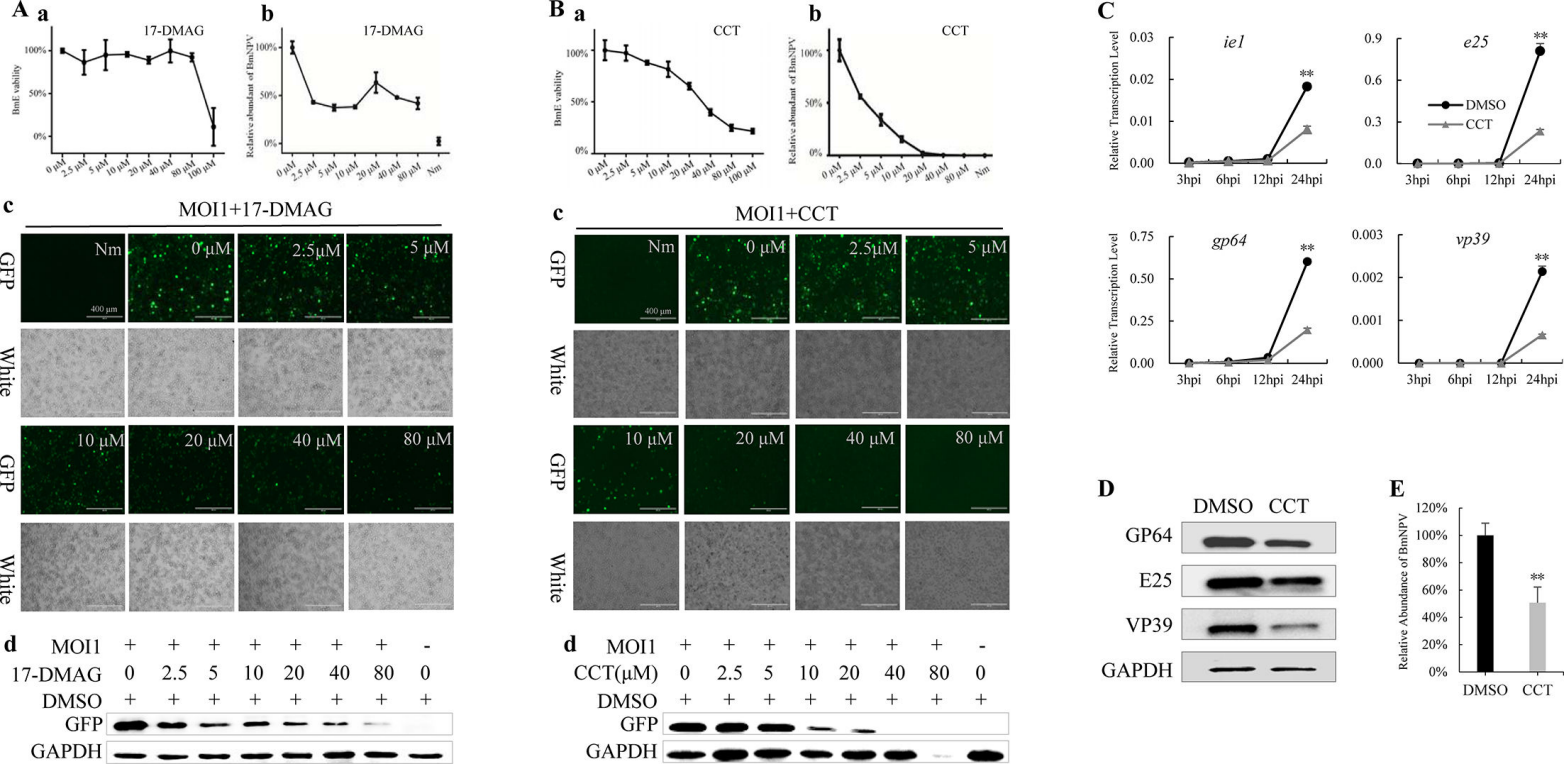
Inhibitory effects that different doses of CCT018159 have on BmNPV. The inhibitory effects that different concentrations of 17-DMAG (**A**) and CCT018159 (CCT) (**B**) have on BmNPV were examined. (a) Cytotoxicity assay: BmE cells were treated with varying drug concentrations, and cell viability was measured using the MTS assay after 72 h. BmE cells were infected with BmNPV-GFP and treated with different drug concentrations. Nm was the uninfected control. (b) Detection of BmNPV content. Total DNA was extracted at 24 h post-infection (hpi). The accumulated viral DNA content was detected via quantitative PCR (qPCR) analysis of the BmNPV gene, *GP41*. The average level of 0 µM was set at 100%, and the other values were standardized against it. (c) Observation of viral fluorescence at 72 hpi. (d) Western blotting of GFP and control GAPDH at 72 hpi. Then, BmE cells were infected with BmNPV-GFP, whereafter 10 µM CCT and control DMSO were added, respectively. (**C**) The qPCR analysis of viral mRNA at 3, 6, 12, and 24 hpi. (**D**) Proteins were extracted at 24 hpi for Western blotting with viral antibodies, and GAPDH was used as a control. (**E**) Accumulated viral DNA content in cells was detected using qPCR. The average level in DMSO at 24 hpi was set at 100%, and the CCT value was standardized against it. Student’s *t*-tests were used to analyze the statistical data. Error bars represent standard deviations. **P < 0.01. The results were confirmed in three independent experiments. MOI, multiplicity of infection.

In the literature, the small molecule compound, CCT018159 (CCT), has been reported to exhibit inhibitory effects on both UAP56 and HSP90 (30), and 17-DMAG is an alternative inhibitor of HSP90. BmE cells were treated with CCT and 17-DMAG at different concentrations (0, 2.5, 5, 10, 20, 40, and 80 µM), and cell viability was assessed using 3-(4,5-dimethyl-2-yl)-5-(3-carboxymethoxyphenyl)-2-(4-sulfophenyl)-2H-tetrazolium (MTS) assays 72 h later, which showed that 17-DMAG did not exhibit significant cytotoxicity at 80 µM (Fig. 1Aa), whereas CCT exhibited dose-dependent cytotoxicity (Fig. 1Ba). After BmNPV infection, different doses of drugs were added, and total DNA was extracted for quantitative PCR (qPCR) detection 24 h later. Results showed that the virus content decreased with an increase in CCT dose (Fig. 1Bb). Fluorescence observation and Western blotting were performed 72 h after infection, showing that virus fluorescence (Fig. 1Bc) and GFP protein content (Fig. 1Bd) decreased with an increase in CCT dose (Fig. 1Bc). However, no notable trends were observed in the 17-DMAG treatment group (Fig. 1Ab–d). These results suggest that CCT targets UAP56 rather than HSP90 to inhibit BmNPV.

After BmE cells were infected with BmNPV, 10 µM CCT was added, and total RNA was extracted at 3, 6, 12, and 24 hpi for qPCR detection. Results showed that the viral mRNA content in the CCT-treated group was significantly lower than that in the control at 24 hpi (Fig. 1C). Total protein and DNA were extracted at 24 hpi, and the viral protein content in the CCT-treated group was found to be significantly lower than that in the control group, as determined via Western blotting (Fig. 1D). Moreover, qPCR showed that CCT caused a significant decrease in the number of viral DNA copies (Fig. 1E). These results suggest that CCT significantly inhibited the proliferation of BmNPV.

### CCT acts during the late gene expression stage of BmNPV

**Fig 2:**
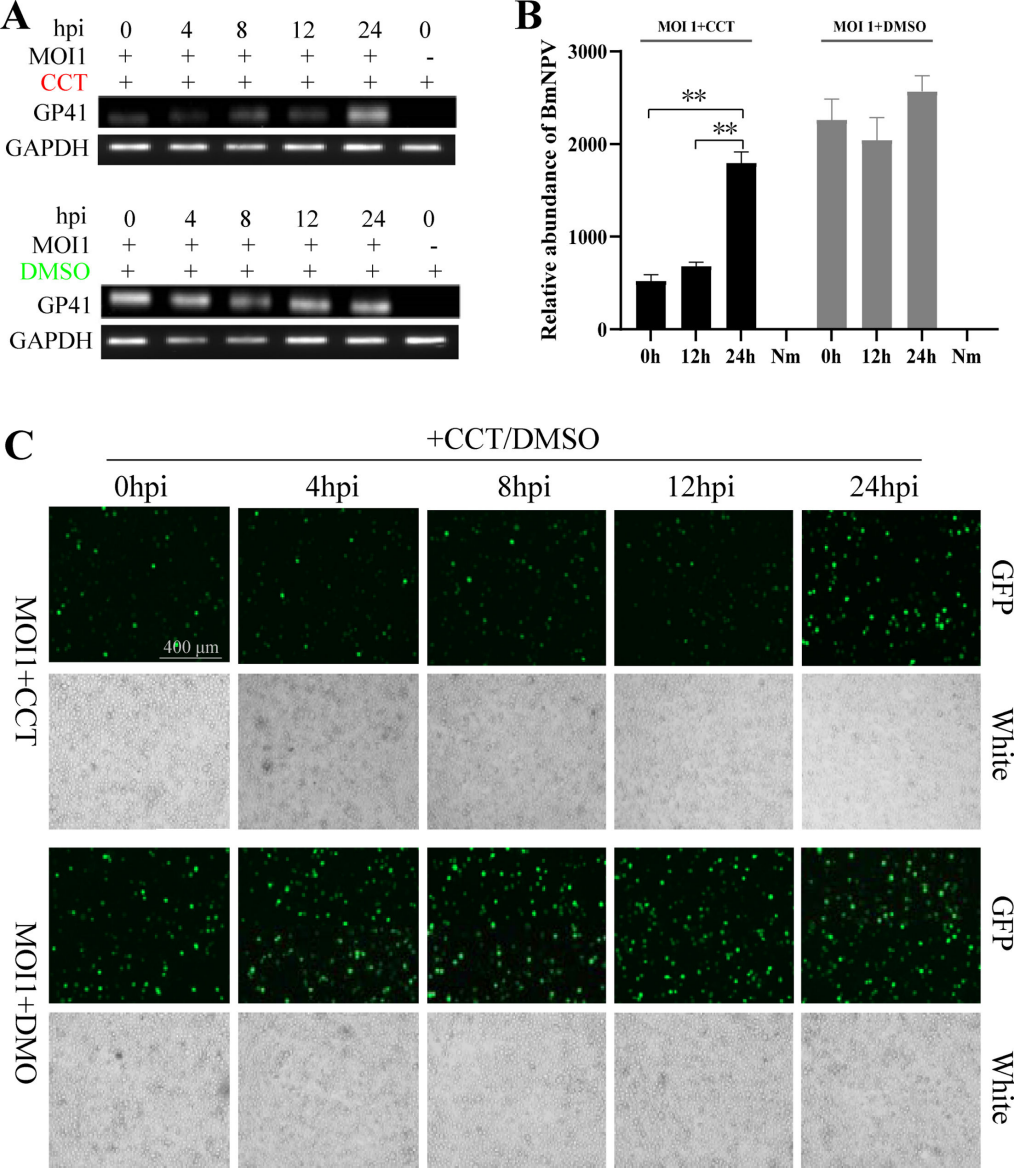
Inhibitory effects of CCT018159 on BmNPV when added at different time points. BmE cells were infected with budded virion-GFP (multiplicity of infection [MOI] = 1), then treated with 10 µM CCT018159 (CCT) and DMSO at 0, 4, 8, 12, and 24 hpi. Total cellular DNA was extracted at 48 hpi. Viral GP41 gene expression was analyzed via reverse transcription-PCR (**A**) and qPCR (**B**) using GAPDH as the internal reference control. Both virions and unencapsidated viral DNA were examined. (**C**) Observation of viral fluorescence at 72 hpi. Nm was the uninfected control. Student’s *t*-tests were used to analyze the statistical data. Error bars represent standard deviations. **P < 0.01. The results were confirmed in at least two independent experiments.

BmE cells were treated with 10 µM CCT at different time points after BV-GFP (a GFP-tagged virus) infection, and dimethyl sulfoxide (DMSO) was added as a control. Total DNA of the cells was extracted 48 h after infection, and the reverse transcription PCR (RT-PCR) results showed that the virus content in the cells was significantly lower than that in the DMSO control group at 0, 4, 8, and 12 h after infection with the addition of CCT (Fig. 2A). The qPCR results showed that the addition of CCT at 0 and 12 h after infection significantly reduced the viral content, and the inhibitory effect exerted by both treatments on BmNPV was nearly identical (Fig. 2B). Fluorescence observation was performed 72 h after infection and showed that virus fluorescence was significantly weaker than that in the DMSO group in cells treated for 0, 4, 8, and 12 h (Fig. 2C). These results suggest that CCT addition within 12 h after infection significantly inhibited BmNPV, whereas CCT addition at 24 hpi had a poor viral inhibitory effect. These findings suggest that the inhibitory effect of CCT on BmNPV occurred mainly during the late viral gene expression stage.

### BmUAP56 interacts with BmNPV 25k

**Fig 3:**
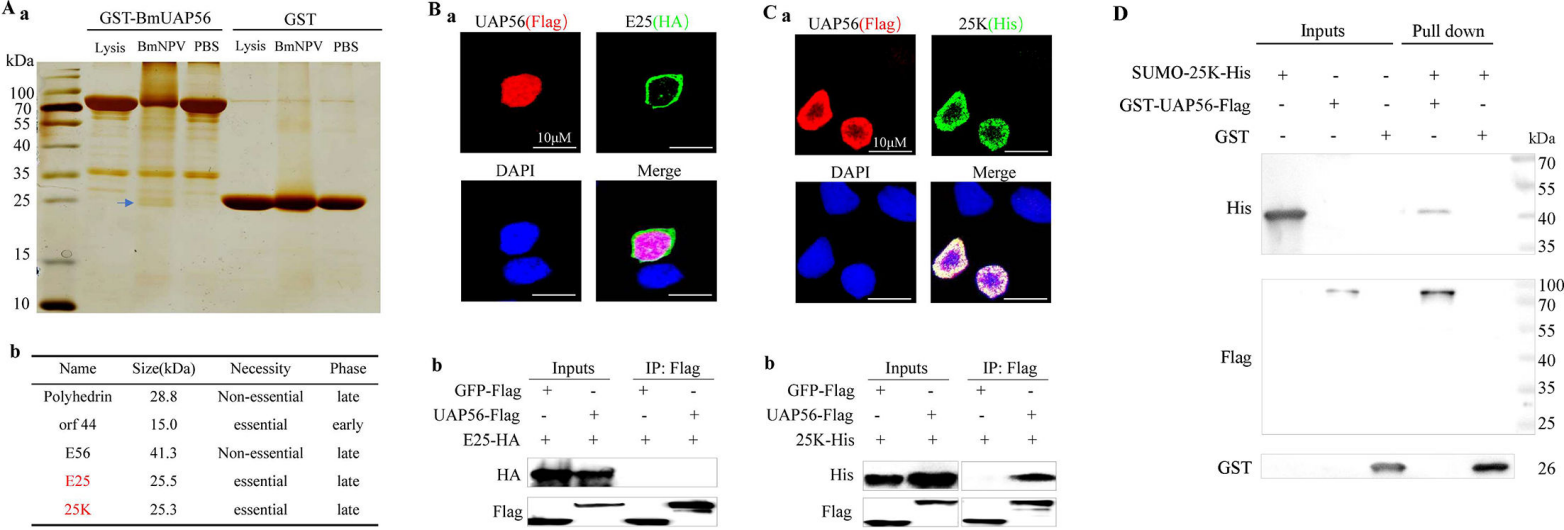
Identification of BmUAP56-interacting viral proteins. (**A**) Pull-down screening of BmUAP56-interacting viral proteins. (a) The midgut protein and protein lysate after ingestion of BmNPV and PBS were taken as inputs, GST-BmUAP56 and GST pulled down, and the specific binding bands (blue arrows) identified via SDS-PAGE detection, which were mined for mass spectrometry analysis, whereafter five viral proteins were identified (b). Identification of whether the viral proteins, E25 (**B**) and 25K (**C**), could bind to BmUAP56. The 1180 basic vector, hr3 enhancer, A4 promoter, and termination signal ser1 were used for construction of transient expression vector 1180-hr3-A4P-UAP56-Flag/E25-HA/25K-His/GFP-Flag-ser1. (a) Subcellular localization: BmE cells were co-transfected with vectors encoding UAP56-Flag with E25-HA and 25K-His, whereafter for immunofluorescence detection. (b) for co-immunoprecipitation (Co-IP) detection, the vectors encoding UAP56-Flag and control GFP-Flag were co-transfected with E25-HA and 25K-His expression vector, respectively. Flag antibody was used for immunoprecipitation (IP), and tag antibodies were used for immunoblotting, respectively. (**D**) Combination *in vitro*. Purified recombinant protein SUMO-25K-His was used as input for the pull-down of GST-UAP56-Flag and GST. Antibodies against His, Flag, and GST were used for immunoblotting. The results were confirmed in at least two independent experiments.

We cloned BmUAP56 and found an 89% amino acid similarity (Fig. S1A) between BmUAP56 and human HsUAP56, which both contain DEXDc and HELIc domains (Fig. S1B). The RT-PCR results showed that BmUAP56 was expressed in silkworms at all developmental stages (Fig. S1C), and qPCR showed that the gene was expressed in all silkworm tissues (Fig. S1D). A prokaryotic expression vector of BmUAP56 was constructed, and its expression was induced at 25°C with 0.1% isopropyl β-d-1-thiogalactopyranoside (IPTG). The target protein band (Fig. S1E) was obvious in the supernatant, and the purified GST-BmUAP56 recombinant protein (Fig. S1FG) was obtained after GST affinity chromatography and molecular sieve and ion exchange purification. At 48 h after the ingestion of BmNPV and phosphate-buffered saline, total midgut protein was extracted as the input, the lysate was used as the control for pull-down of GST-BmUAP56 and the control GST. Electrophoresis showed that BmNPV had a specific binding band with GST-BmUAP56, with a size of approximately 25 kDa (Fig. 3Aa). The band was mined for mass spectrometry analysis (Table S1), and five viral proteins were identified: *polyhedrin*, *orf44*, *E25*, *E56*, and *25K* (Fig. 3Ab). Among them, only E25 and 25K were late essential genes of BmNPV, which were thus cloned to construct incremental expression vectors. BmE cells were co-transfected with vectors encoding HA-tagged E25 and His-tagged 25K with BmUAP56-Flag. Immunofluorescence showed that E25 and BmUAP56 proteins localized to different locations in the cell (Fig. 3Ba), whereas 25K co-localized with BmUAP56 in the nucleus (Fig. 3Ca). The co-immunoprecipitation (Co-IP) results showed that E25 did not bind to BmUAP56 or control GFP (Fig. 3Bb), but 25K specifically bound to BmUAP56 (Fig. 3Cb). These results suggest that BmUAP56 binds to the late essential protein, 25K, of the baculovirus. The recombinant protein GST-UAP56-Flag and SUMO-25K-His were purified (Fig. S1H). Pull-down results indicated that SUMO-25K bound to GST-UAP56 but not GST (Fig. 3D), and SUMO-25K but not SUMO bound to GST-UAP56 (Fig. S2), suggesting that BmUAP56 and 25K are directly interacted.

### 25K binding to BmUAP56 promotes nuclear export of viral mRNA

**Fig 4:**
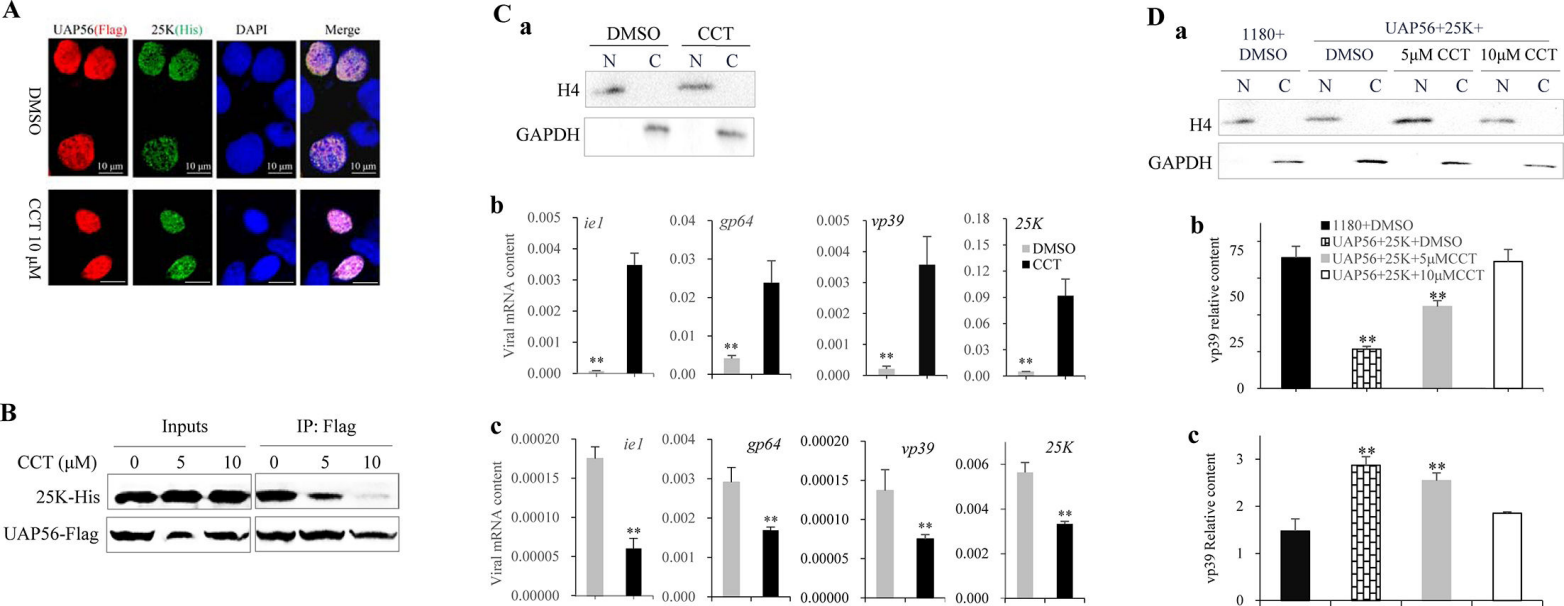
CCT018159 blocks the binding of UAP56 to 25K and inhibits the export of viral mRNA nuclei. (**A**) CCT018159 (CCT) does not affect the co-localization of BmUAP56 with 25K. BmE cells co-transfected with UAP56-Flag and 25K-His expression vectors were supplemented with 10 µM CCT or DMSO and subjected to immunofluorescence. (**B**) CCT blocks the binding of UAP56 to 25K. BmE cells co-transfected with 25K-His and UAP56-Flag expression vectors were added with 0, 5, and 10 µM CCT, whereafter Co-IP detection was performed. (**C**) CCT inhibits the nuclear export of viral mRNA. (a) DMSO and 10 µM CCT were added to BmE cells infected with BmNPV, and the nucleus (N) and cytoplasm (C) were separated and detected with antibodies against H4 and GAPDH. (b) Nuclear and (c) cytoplasmic RNA was extracted for qPCR to determine the mRNA content of the virus. (**D**) Incremental expression of UAP56 and 25K promoted the nuclear export of viral mRNA. Vector 1180-hr3-A4P-VP39-ser1 was constructed for transient expression of viral gene VP39. BmE cells were co-transfected with vectors encoding VP39, UAP56, and 25K, and then 0, 5, and 10 µM CCT were added, respectively. The co-transfected cells of basic 1180 and VP39 expression vectors were added with DMSO as a control. (a) Western blotting detection was performed after nucleoplasm isolation. The qPCR assay was used to detect the mRNA content of VP39 in the nucleus (b) and cytoplasm (c). Student’s *t*-tests were used to analyze the statistical data. Error bars represent standard deviations. **P < 0.01. The results were confirmed in three independent experiments.

BmE cells were co-transfected with UAP56-Flag and 25K-His expression vectors and treated with 10 µM CCT for 48 h. CCT did not affect the co-localization of BmUAP56 and 25K proteins (Fig. 4A). Co-IP experiments showed that CCT inhibited the binding of BmUAP56 to 25K, and the inhibitory effect increased with an increase in drug concentration (Fig. 4B). After BmE cells were infected with BmNPV, 10 µM CCT was added, and the cells were extracted at 24 hpi for nucleoplasmic isolation. Antibodies for H4 and GAPDH were detected in the nucleus and cytoplasmic protein samples, respectively, indicating that nucleoplasmic isolation was successful (Fig. 4Ca). The qPCR detection results showed that the mRNA content of the virus in the nucleus of the CCT treatment group significantly increased (Fig. 4Cb). The opposite result was observed in the cytoplasm (Fig. 4Cc). The transient expression vector of viral gene VP39 was constructed and transfected into BmE cells, and nuclear and cytoplasmic RNA was extracted after nucleoplasm isolation (Fig. 4Da). The mRNA content of VP39 in the nucleus of BmE cells co-transfected with VP39, UAP56, and 25K expression vectors was significantly lower than that in the control group (only basic 1180 and VP39 expression vectors were transfected) (Fig. 4Db), while a contrary result was detected in the cytoplasmic RNA (Fig. 4Dc). These trends could be reversed by the addition of CCT (Fig. 4Db and c). These results suggest that 25K binds to BmUAP56 and promotes the nuclear export of viral mRNA and that CCT inhibits this process by blocking the binding of BmUAP56 and 25K.

## DISCUSSION

Viruses commonly hijack key genes in hosts to achieve efficient infection. UAP56 is highly conserved in different species and has important biological functions. Therefore, it has become a target of viral regulatory hosts (31). Our study revealed that UAP56 is involved in baculovirus infection.

CCT targets UAP56, and 17-DMAG is an alternative inhibitor of HSP90 (30). CCT inhibited the proliferation of BmNPV, and the inhibitory effect increased with an increase in drug concentration; however, 17-DMAG exhibited no similar phenomenon (Fig. 1). The addition of CCT within 12 hpi significantly inhibited viral proliferation (Fig. 2), indicating that CCT exerts its function in the late gene expression stage of BmNPV. Therefore, if a BmNPV protein binds to BmUAP56, it should be considered as a late essential gene of the virus. Through protein expression, pull-down, immunofluorescence, and Co-IP analysis, the late essential gene of BmNPV, 25K, was demonstrated to directly bind to BmUAP56 (Fig. 3), whereafter they promote the nuclear export of viral mRNA (Fig. 4). CCT can block the binding of BmUAP56 and 25K, resulting in the abnormal accumulation of viral mRNA in the nucleus, causing a decrease in viral content in the cell, and ultimately inhibiting viral proliferation.

As a central hub of host RNA metabolism, UAP56 plays a key role in mRNA transcription, splicing, and nuclear export (32). It is one of the key targets of virus-host interactions and is involved in the infection of a variety of viruses. After influenza virus infects the host, the viral NP protein directly binds to UAP56 to maintain stability of the vRNA-NP complex, promote the formation of ribonucleoproteins, and regulate viral RNA synthesis through helicase activity (33). The Rev protein of HIV-1 binds to UAP56 and assists in the export of unspliced/partially spliced mRNA via the CRM1 noncanonical pathway, which may be involved in helicase-dependent gRNA dimerization (34). The SM protein of Epstein-Barr virus binds to UAP56 to promote the stability and export of mRNA during the late stage of the virus (35). The ICP27 protein of herpes simplex virus recruits UAP56 and Aly/REF to bypass host splicing checkpoints and directly exports incompletely spliced viral mRNA (36, 37). Our results suggest that the baculovirus 25K protein binds to UAP56 to promote the nuclear export of viral mRNA, thereby ensuring viral proliferation. The baculovirus genome does not contain introns, which would necessitate the hijacking of UAP56 for efficient nuclear export of viral mRNA. Hijacking UAP56 may be a conservative strategy used by viruses to regulate their hosts, although its role varies among different viral infections.

The 25K protein plays an important role during multiple stages of baculoviral infection. Previous studies have found that 25K regulates both replication and structural stability, as observed through viral gene knockout, Co-IP, cryo-electron microscopy, and other methods. Baculovirus 25K forms a functional replication complex with DNA polymerase, helicase (P143), and the LEF-1/2 cofactor (38), which plays a scaffolding role in the initial stage of DNA replication; deletion of 25K leads to replication arrest, as well as loose nucleocapsid structure and VP39 arrangement (39). Notably, the function of 25K is spatiotemporally specific: early deletions result in the defective generation of ODVs, whereas late deletions do not affect BV release (40). Transcriptome analysis revealed that 25K balances the viral transmission strategy by regulating the expression of ODV genes, such as p74, and BV genes, such as gp64 (41, 42). In the present study, the binding of viral proteins to UAP56 was blocked by inhibitors without missing 25K, and 25K was found to demonstrate a new function of hijacking UAP56 to promote the nuclear export of viral mRNA. Deletion of 25K results in higher levels of BV (43), but inhibition of binding with UAP56 appears to have the opposite effect. Key sites for the binding of UAP56 to 25K could be identified in future experiments and targeted through gene editing to enhance the antiviral capacity of silkworms.

In conclusion, we confirmed that the baculovirus 25K protein hijacks host UAP56 to facilitate the nuclear export of viral mRNA and consequently cause infections. This research not only deepens our understanding of the transcription and translation mechanisms used by baculoviruses but also provides potential targets for antiviral research.

## MATERIALS AND METHODS

### Silkworms, cells, and viruses

The *B. mori* Dazao strain was maintained at the Silkworm Gene Resource Bank of Southwest University (Chongqing, China). BmE cells were cultured at 27°C. BmNPV and GFP-expressing BmNPV were collected from the hemolymph of infected silkworm larvae and BmE cells, respectively.

### Viral challenge and toxicity assay

BmE cells were seeded in 24-well plates and infected with BmNPV at a multiplicity of infection of 1 by adding 0.1 µL viral suspension per well, followed by incubation for 1 h. After infection, the viral medium was replaced with a drug-containing medium, and the cells were cultured for an additional 48 h. Genomic DNA was extracted using an OMEGA kit, and viral proliferation quantified via qPCR targeting the late viral gene, *GP41*, and the reference gene, *GAPDH*. Viral fluorescence was observed under green and white light at 72 hpi. The total protein was extracted for Western blotting using antibodies against GFP and GAPDH at 72 hpi. Student’s *t*-test was used to analyze the statistical data.

### Drug cytotoxicity assay

Cell viability was measured using an MTS-based CellTiter 96 AQueous One Solution Cell Proliferation Assay (Promega). Cells were seeded in 96-well plates with 80 µL cell suspension or medium per well (four replicates per drug concentration), alongside four background control wells containing medium only. Each well received 20 µL drug-supplemented complete medium, with DMSO as a control. After 72 h at 27°C, 20 µL Cell Titer 96 AQueous One Solution Reagent was added, followed by a 3 h incubation in the dark. Absorbance at 490 nm was measured using a microplate reader, and background-subtracted values were used to calculate the relative cell viability as a percentage of the control group.

### Plasmid construction, protein expression, and purification

A PGEX-4T-BmUAP56 vector for the fusion expression of GST-BmUAP56 was constructed and transformed into competent cells. Single colonies were expanded in culture for 4–6 h at 37°C and 220 rpm, followed by induction with 0.1% IPTG under varying conditions (4 h at 37°C, 10 h at 25°C, or 20 h at 16°C). Uninduced controls were cultured at 37°C for 4 h. Cells were harvested using centrifugation (8,000 × *g*, 5 min), resuspended in equilibration buffer A, and lysed via sonication. The lysate was loaded onto a GST column pre-equilibrated with buffer A, and the bound proteins were eluted using 1 and 10 mM reduced glutathione. The column was washed and stored in 20% ethanol at 4°C.

### Pull-down and mass spectrometry

Fifth-instar Dazao silkworms were orally infected with BmNPV at a density of 1 × 10^9^ polyhedra per larva. Midguts were dissected at 48 hpi, and proteins were then extracted. GST beads (30 µL) were mixed with 1 mL equilibration buffer A, centrifuged, and incubated with 0.9 mg purified protein (double the GST bead-binding capacity). After overnight rotation at 4°C, beads were washed five times with buffer A. Bound proteins were eluted, resolved via sodium dodecyl sulfate-polyacrylamide gel electrophoresis (SDS-PAGE), and specific bands were excised for mass spectrometry analysis.

### Cellular fluorescence localization

The expression vectors 1180-hr3-A4P-UAP56-Flag/E25-HA/25K-His/GFP-Flag-ser1 were constructed using 1180 basic vector, hr3 enhancer, A4 promoter, and termination signal ser1 (14). BmE cells co-transfected with vectors encoding UAP56-Flag and 25K-His were fixed with 4% paraformaldehyde, permeabilized with 1% Triton X-100, and blocked with immunofluorescence blocking buffer. Primary antibodies (His-Tag [D3I1O] XP Rabbit mAb and DYKDDDDK Tag [9A3] Mouse mAb, 1:200 dilution) were applied overnight at 4°C, followed by fluorescent secondary antibodies (1:500 dilution) and DAPI (4′,6-diamidino-2-phenylindole) counterstaining. The coverslips were mounted with ProLong Diamond Antifade mountant and imaged using a ZEISS LSM 880 confocal laser scanning microscope (Carl Zeiss, Oberkochen, Germany) under the appropriate laser channels.

### Co-IP and Western blotting

Cells were harvested 48 h post-transfection and lysed for Co-IP using the EZview Red ANTI-FLAG M2 Affinity Gel. Beads were washed with NP-40 buffer, and bound proteins were resolved via SDS-PAGE, transferred to polyvinylidene fluoride membranes, and then blocked overnight. The membranes were probed with His-Tag (D3I1O) and DYKDDDDK Tag (D6W5B) antibodies (1:2,000 dilution), followed by horseradish peroxidase-conjugated goat anti-rabbit IgG (1:5,000). Signals were detected using the SuperSignal Western Blot Substrate and visualized with an SH-Advance523 imaging system.

### Combination experiment *in vitro*

The pSUMO vector contains a SUMO tag (44). A pSUMO-25K vector for the fusion expression of SUMO-25K-His was constructed. The recombinant protein GST-UAP56-Flag and SUMO-25K-His were constructed through prokaryotic expression and protein purification. SUMO-25K-His and SUMO-His were used as inputs for the pull-down of GST-UAP56-Flag and GST. Antibodies against His, Flag, and GST were used for Western blotting.

### Nuclear-cytoplasmic fractionation, RNA extraction, and qPCR

Nuclear and cytoplasmic fractions were isolated using the PHYGENE Nuclear/Cytosol Fractionation Kit (PH1466). Total RNA was extracted with the Eastep Super Total RNA Extraction Kit. The qRT-PCR assay was performed using NovoStart SYBR qPCR Super Mix Plus on a qTOWER3/G system (Analytik Jena, Jena, Germany). qPCR data were used for analyzing the relative changes in gene expression using the 2^−ΔΔCt^ method.

## Data Availability

The raw qPCR data are available in the BioProject database (https://ngdc.cncb.ac.cn/bioproject/browse/) under accession number PRJCA045606. Additional data are available on request from the authors.

## SUPPLEMENTAL MATERIAL

Supplemental figures jvi.01248-25-s0001.docx Fig. S1 and S2.

Table S1 jvi.01248-25-s0002.xlsx Identification of viral proteins by mass spectrometry.

ASM does not own the copyrights to Supplemental Material that may be linked to, or accessed through, an article. The authors have granted ASM a non-exclusive, world-wide license to publish the Supplemental Material files. Please contact the corresponding author directly for reuse.
