# Supplementary material for: Baculovirus 25K hijacks host UAP56 to facilitate nuclear export of viral mRNA in insect cells
Source: J Virol. 2025 Sep 23;99(10):e01248-25. doi: 10.1128/jvi.01248-25 (PMC12548385; doi:10.1128/jvi.01248-25)
Supplement: Supplemental figures — Fig. S1 and S2. [file jvi.01248-25-s0001.docx]

**
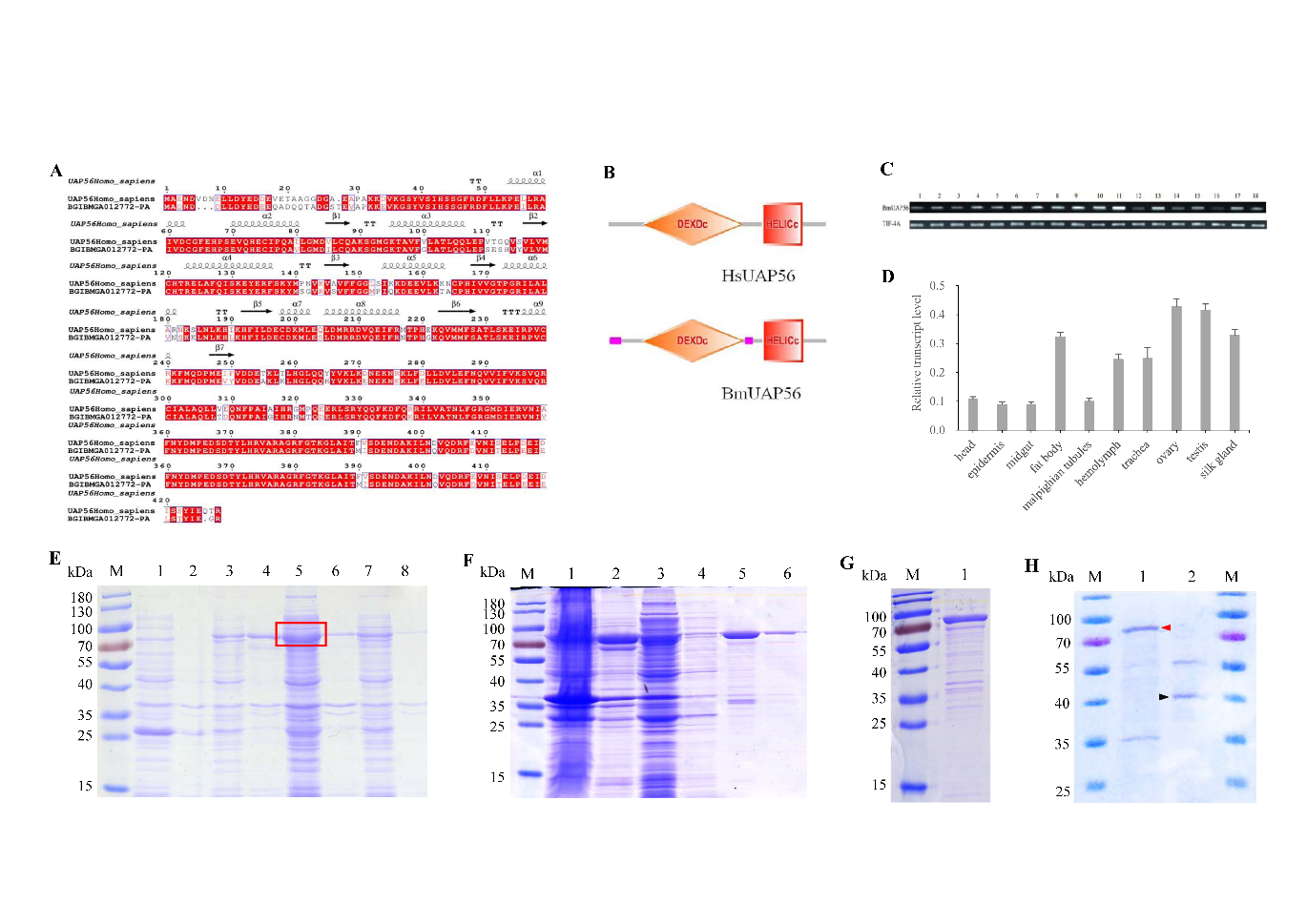
**

**Figure S1.**

(A) Amino acid sequence alignment of the silkworm and human UAP56. The NCBI reference sequence of *Homo sapiens* UAP56 (HsUAP56) and BmUAP56 (BGIBMGA012772) was NP_004631.1 and XM_004928946.4, respectively. (B) UAP56 domain of the silkworm and human, both contain DEXDc and HELICc domains. Spatiotemporal expression pattern analysis of BmUAP56. (C) Reverse transcription-PCR detection of the expression spectrum during different periods: 1, egg-2th-day; 2, egg-2th-day; 3, egg-2th-day; 4, egg-2th-day; 5, hatch; 6, Ⅰmolt; 7, Ⅱinstar; 8, Ⅱmolt; 9, Ⅲinstar; 10, Ⅲmolt; 11, Ⅳinstar; 12, Ⅳmolt; 13, Ⅴinstar; 14, pupa-2th-day; 15, pupa-4th-day; 16, pupa-6th-day; 17, pupa-8th-day; 18, moth. (D) The quantitative PCR assay was used to detect the expression of tissues. (E) Prokaryotic expression of the GST-BmUAP56 fusion protein. Lanes: 1–2: empty vehicle control (37°C); 3–4: GST-BmUAP56 (37°C, 0.1% β-d-1-thiogalactopyranoside [IPTG], supernatant, precipitation); 5–6: GST-BmUAP56 (25°C, 0.1% IPTG, supernatant, precipitate); 7–8: GST-BmUAP56 (16°C, 0.1% IPTG, supernatant, precipitate). (F) Affinity chromatography purification of GST-BmUAP56: GST column purification procedure. Lane 1, cleavage supernatant; 2, precipitation; 3, flow-through fluid; 4, equilibration buffer; 5, 1 mM glutathione; 6, 10 mM glutathione; M, protein ladder. (G) Molecular sieve chromatography purification of GST-BmUAP56: separation of protein peak components collected after purification (lane 1); M, protein ladder. (H) Purified recombinant protein. Lane 1, GST-UAP56-Flag (red arrow); 2, SUMO-25K-His (black arrow); M, protein ladder. The results were confirmed in at least two independent experiments.


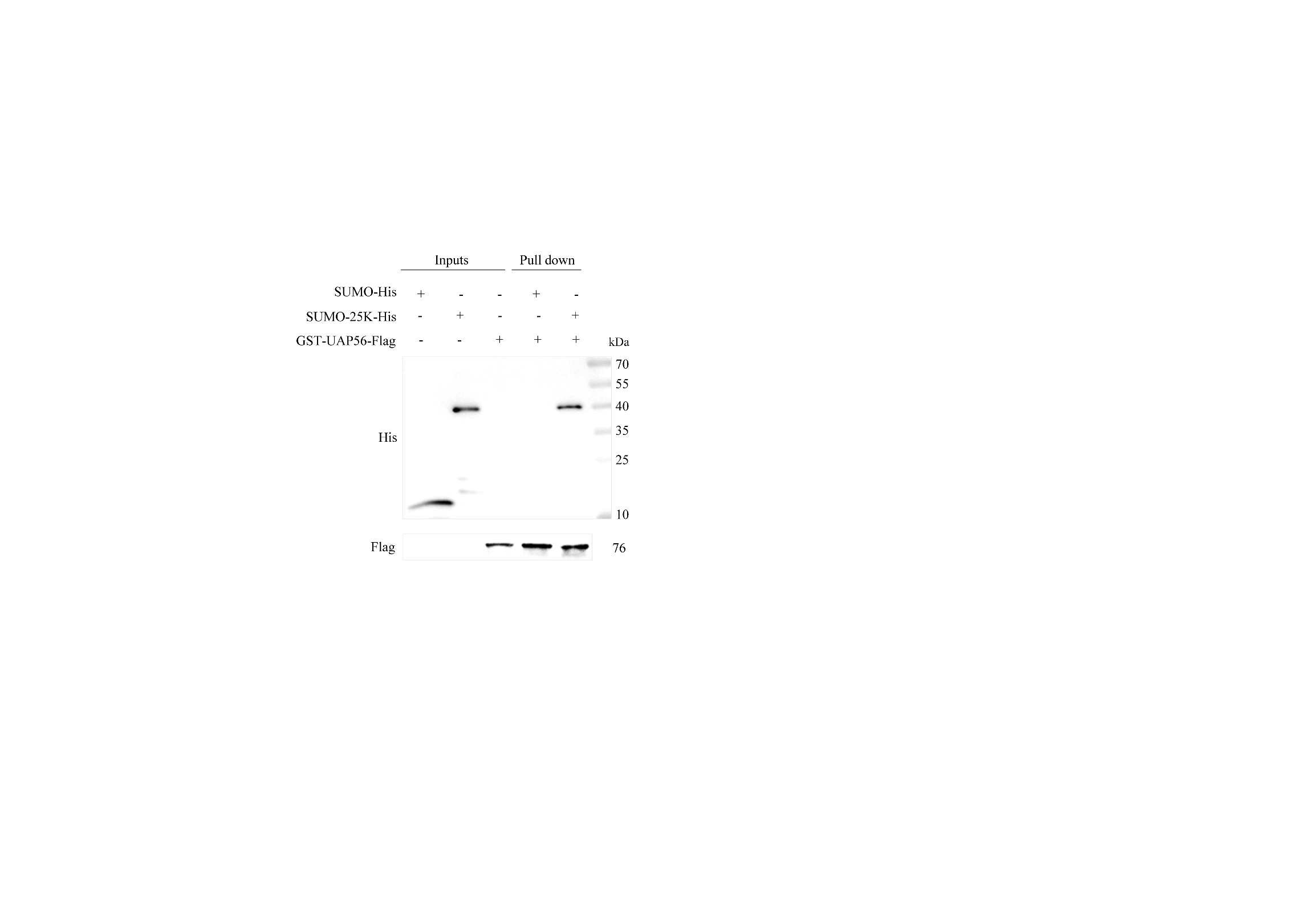


**Figure S2.**

Purified recombinant protein SUMO-25K-His and control SUMO-His were used as inputs for the pull-down of GST-UAP56-Flag. Antibodies against His and Flag for immunoblotting. The results were confirmed in at least two independent experiments.
